# Finding possible pharmacological effects of identified organic compounds in medicinal waters (BTEX and phenolic compounds)

**DOI:** 10.1007/s00484-019-01808-9

**Published:** 2019-10-31

**Authors:** István Szabó, Csaba Varga

**Affiliations:** Department of Environmental Health, Institute of Public Health Medicie, Medical School, University of Pécs, 12. Szigeti St., Pécs, H7624 Hungary

**Keywords:** Thermal water, Organic compounds, Medicinal water, Therapeutic effect, Organic theory, BTEX, Phenolic compounds

## Abstract

Medicinal thermal waters consist of a mixture of different organic and inorganic compounds. Traditionally, these waters are only characterized and classified by their inorganic composition; however, the bioavailability of the majority of these inorganic compounds is limited. Many authors investigate the organic fractions of thermal waters. These authors propose that these compounds have a potential effect on health. To elucidate the underlying mechanisms, it is crucial to know the composition of the organic fractions. The absorption of these compounds on intact skin or mucosa is notable. Some of them have local anaesthetic effect or affect receptors in the central nervous system. In the knowledge of the chemical composition, we are able to estimate the possible pharmacological effect or might be able to assess possible toxicity risks. In the present article, we aim to review possible health effects of two of the identified organic fractions: benzene and alkylbenzenes and phenolic compounds that might correlate with the therapeutic effect on rheumatological or other diseases.

## Introduction

The medical treatment of pain in musculoskeletal or rheumatic disorders mostly involves analgesic drugs. Despite their potency, other additional methods are often needed. Balneotherapy is a well-known and effective auxiliary treatment without severe side effects (Bender et al. 2005). Many of these well-known waters are proven to have a positive effect on certain diseases, such as rheumatological disorders like fibromyalgia or dermatological problems; thus, they are referred to as medicinal waters (Fioravanti et al. 2018; Péter et al. 2017; Varga 2012a). Many of these thermal waters have been used for centuries. Oral traditions and observations attest their effect. Organizing and controlling a balneotherapy study is problematic. Furthermore, research funding is insufficient. A traditional knowledge of healing effect is rarely supported by randomised controlled trials (Bender et al. 2005). Following the concept of evidence-based medicine, Bender et al. (2014) proposed evidence-based balneotherapy. From the pharmacological point of view, high-quality human evidence is needed to prove the effectiveness of medicinal therapies. This evidence relates to the exact formulation, exposure and pharmacokinetics that were investigated in the precise clinical trial. In classical pharmacology, the precise pharmacodynamic mechanisms of some of the most prevalent medicine have not been elucidated yet (for example lithium) (Malhi et al. 2013). In clinical trials, only the efficiency needs to be proved. Furthermore, some compounds have been used for centuries and marketed as medicine for more than a century. As the evidence-based medicine concept and rigorous efficiency and toxicological trials appeared in the middle of the twentieth century, these medicines are without these evidences (Sur and Dahm 2011). As balneotherapy has been used for millennia, the therapeutic use of medicinal waters belongs to the same category (Gutenbrunner et al. 2010; Bender et al. 2005, 2014). The last two decades abound in evidence from randomized controlled trials and their meta-analyses. In these studies, usually a selected method of balneology was investigated mainly in rheumatic diseases. These pieces of evidence confirm the experience-based use of balneotherapy (Bender et al. 2014; Fioravanti et al. 2017). Medical significance of the organic fractions of natural waters is still poorly understood. The classical hypothesis of the categorisation of spa waters is based on analysing the inorganic contents and marks only the total organic matter content (Varga 2010). The reason for this type of classification is that inorganic quantitative and qualitative analyses are easy to conduct. From the medical point of view, the utilization of these classifications is faulty. Firstly, the dermal bioavailability of most of these salts is poor considering that most of the balneotherapy involves immersion in medicinal water or peloid packs (Shani et al. 1985; Gröber et al. 2017). Secondly, the organic matter content is outstanding concerning the quantity and the diversity of the compounds too (Kárpáti et al. 1999; Fekete et al. 2009). Furthermore, the dermal intake of many of these compounds (phenolics, BTEX compounds) is remarkable (Lim et al. 2014; Monteiro-Riviere et al. 2001).

In the recent past, several authors proposed that medicinal waters, especially thermal spa and hot spring waters, contain a high variability of organic components with possible biological effects that might contribute to the healing mechanisms (Fekete et al. 2012; González-Barreiro et al. 2009; Varga 2012b). Varga and his coworkers have been investigating both benign (curative) and disadvantageous (toxic) effects of the organics in thermal waters. They claim that many of these organic compounds are highly bioactive in the scope of other scientific fields. Therefore, these fractions cannot be neglected when discussing balneology (Varga et al. 1992; summarized in Varga 2012a). González-Barreiro et al. also identified organic compounds in several Spanish traditional medicinal waters that “are responsible for the biological activity of thermal waters” (González-Barreiro et al. 2009). Fekete et al. found great importance of analysing the organic content of thermal waters, because “thermal waters are used for balneologic purposes and organic compounds may contribute to the curative power” (Fekete et al. 2012). Hanzel et al. conducted the first clinical trial that investigates organic content separately. Organic fraction of Szigetvár medicinal water was separated and later diluted by heated tap water. As a result of this process, an artificial medicinal water was created without the original inorganic content. The investigated parameters for osteoarthritis were significantly better compared with that of tap water control, which proves the beneficial effect of the organic matter (Hanzel et al. 2019). Analytical researches provide us with the actual organic matter composition. However, selecting a single compound or groups of chemicals from this entity and assign pharmacological mechanisms based on available evidences is only of theoretical interest. In the absence of proper pharmacokinetic data and models from balneotherapy, this is impossible. In most cases, the applied concentrations do not match the concentration found in thermal waters. When searching for evidence in balneotherapy, the best strategy is to conduct human clinical trials testing the whole thermal water or selected fractions of the thermal water.

This review aims to gather evidence where the effect of the organic compounds matches the effect of balneotherapy. In the present article, we select two groups of organic matter, benzene/alkylbenzenes and phenolic compounds, to review possible pharmacological effect or mechanisms of action that might correlate to the pharmacological effect of medicinal waters. These pharmacological effects or mechanisms of action might be in the focus of future balneopharmacological studies.

## Organic chemical analysis of thermal waters

The first organic analytical investigations in Hungary were carried out by Agyagási. He identified open-chain olefins in thermal waters (Agyagási 1983). Since the 1990s, several researchers have investigated the organic content of thermal waters of the Carpathian Basin (Vető and Bertalanné Balogi 1994; Kárpáti et al. 1999; Fekete et al. 2009; Fekete 2013). The balance of organic and inorganic components was studied in thermal waters. The decomposition of organic matter produces small molecular-sized aromatic compounds and also influences the amounts of inorganic components (Fekete et al. 2009). The decomposition of organic matter is strongly linked to a temperature threshold. As the temperature of the water increases, organic matter decomposes and the concentration of dissolved aromatics (benzene homologues, phenols) increases. Depending on the temperature, different homologue series appear, aromatic hydrocarbons dominate at a threshold of 80 °C, phenols at 90 °C and in those hotter than 90  C fatty acids can be identified (Fekete et al. 2012).

Thermal waters were classified to phenol free and phenolic waters. Phenols are the predominant aromatic compounds of hotter waters, while benzene and alkylbenzenes dominate in lower concentrations (Kárpáti et al. 1999; Fekete 2013). The identified organic matter (especially BTEX (benzene, toluene, ethylbenzene, xylenes) compounds) can originate from environmental pollution caused by petrol consumption or the co-existence of underground oil reservoirs. In the reviewed articles, the investigated thermal waters did not have any connection with oil pollutants or natural oil sources (Kárpáti et al. 1999; Fekete et al. 2009; Fekete 2013).

In several thermal waters, phenol and simple phenol derivatives with ethyl and methyl substituents were identified. The concentrations of these compounds range from 1 μg/L up to 1–2 mg/L. Among many other aromatic compounds, these phenolic compounds originate from humic substances or lignite, which decompose to carbon dioxide, hydrocarbon gases and water soluble aromatic compounds (alkylbenzenes, polycyclic aromatic hydrocarbons (PAH), heteroaromatic compounds). The main groups of organic matter were humic substances, benzene and alkylbenzenes, PAH and phenolic compounds (Fekete 2013). Kompanichenko et al. analysed the thermal waters of the Uzon caldera and the Mutnovskii geothermal area. The researchers identified the following organic chemical groups: alkanes, aromatic hydrocarbons, ketones, alcohols (Kompanichenko et al. 2016). Szabó et al. reviewed the possible pharmacological effect of the organic matters identified in Kakasszék medicinal water. Kakasszék is a phenolic type of water. The main compounds were simple phenol derivatives (Szabó et al. 2013).

González-Barreiro analysed water samples from four open water springs of Galicia thermal spa region. They identified several chemical families including N-compounds, heterocyclics, amines, aromatic hydrocarbons, esthers, ketones and phenols. Volatile aromatic hydrocarbons benzene, ethylbenzene and xylenes were detected in all of the samples; however, the concentrations were relatively low. Such compounds are the product of thermal degradation of essential organic chemicals such as carotenoids or amino acids. Several alkylphenols were also identified in all samples. The concentrations ranged from 9 to 15%. These compounds originate from lignin-rich aquifers or maturation of thermal waters (González-Barreiro et al. 2009).

## Benzene and alkylbenzenes

Precise kinetic data from inhalation and dermal absorption of alkylbenzenes are not available from the field of balneology. BTEX compounds as toxic exposure are prevalent in petrol industry. Exposure to BTEX compounds in petrol industry workers was measured along with the concentration of chemicals in ambient air. Mean urinary volatile organic compound (VOC) concentrations ranged up to 11.83 μg/L for highly exposed loading workers and 2.58 μg/L for office workers (Heibati et al. 2018). Biomonitoring after inhalation exposure of mixed BTEX compounds is modelled and is available (Marchand et al. 2015). Urinary VOC concentration biomonitoring could be a possible measure to BTEX exposure during balneotherapy. As benzene and alkylbenzenes are prevalent (eco)toxic compounds, many authors reviewed the toxicology of them (IARC Monograph vol. 120 (2017); Bolden et al. 2015; Heibati et al. 2018). Many drug users (even heavy drug users—poly drug use) chose solvents or glues to inhale their volatile compounds for the dazzling sensation and euphoric properties. These are mainly caused by benzene derivatives, so toluene is also extensively reviewed (Cruz et al. 2014). Studies have shown that neurotransmitter systems (glutamatergic, opioidergic, GABAergic (gamma-aminobutyric acid)) are affected by benzene derivatives (Páez-Martinez et al. 2008). VOCs and toluene have nonselective actions on several voltage-gated ion channels and neurotransmitters in the central nervous system (CNS). They disturb the native balance of ligand-gated ionic channels found in the CNS. Toluene inhibits some excitatory ion channels such as the nicotinic acetylcholine receptors (nAChRs) and N-methyl-d-aspartate (NMDA) glutamate receptors. Toluene potentiates the inhibitory channels, such as serotonin, glycine and GABAA receptors (Bale et al. 2007; Woodward and Beckley 2014). According to the ion channel hypothesis, the acute effect of toluene and VOCs is due to affecting specific ion channels of targets in the CNS. Despite the knowledge of these mechanisms, their overall effect and relative importance is still under investigation (Bale et al. 2007; Evans and Balster 1991; Woodward and Beckley 2014). It is not just a coincidence that in mood disorders, the disturbance of norepinephrine, dopamine, acetylcholine, glutamate and GABA receptors were reported as biological abnormalities, thus basing the neurotransmitter theories (Hassler 2010). Positive modulation of the GABAA receptor is the common mechanism of certain CNS depressants. Solvents like benzene and toluene showed anxiolytic-like effect when tested in animal experiments. This effect is based on the positive modulation of GABAA receptors, which is a common mechanism of action with other CNS depressants as well (Bowen et al. 2006). Certain antidepressant drugs increase noradrenaline and serotonin levels in corresponding brain areas. Many solvents, like toluene, have the same effect. The antidepressant effect of toluene was tested on two animal models capable of screening possible antidepressants. Toluene exerted concentration-dependent antidepressant effect (Cruz et al. 2009). Páez-Martinez et al. investigated a single toluene exposure in connection with morphine administration. When given simultaneously, toluene counteracted the effect of morphine, thus exerting a nociceptive effect. Mu-opioid receptor binding levels were also investigated in different parts of the brain of mice exposed to a single dose of toluene (4000 ppm). The binding levels significantly decreased. Based on these results, toluene exposure might have an effect on analgesic effect of opioids (Páez-Martinez et al. 2008).

## Phenolic compounds

Aromatic compounds have been used in medical sciences for centuries. Phenol was introduced by Lister for disinfectant or antiseptic purposes in medicine. The tissue necrotic effect of phenol is well known (Wood 1978). Since the discovery of Lister, the antiseptic use of phenol faded. It has been replaced with safer and more potent substances concerning antimicrobial activity (Davidson and Branden 1981). The domestic use of concentrated phenol solutions for disinfection is obsolete in the scope of chemical safety rules of today. However, some countries are still manufacturing such products. These products are used for veterinarian, household cleaning and human delousing purposes. Even in the near past chemical burns caused by such products were reported (Vearrier et al. 2015). Cosmetic surgeons use a type of deep chemical peel called phenol face peeling. They apply phenol solution on the face to remove imperfections such as acne scars, wrinkles and blotchiness (Park et al. 2007). Botta et al. reviewed several case reports from cosmetic surgeons and the incidence of arrhythmias in some cases was as high as 23%. Even that time this was considered unacceptable. Therefore, they proposed a new protocol involving cardiac monitoring, fluid loading and the use of prophylactic antiarrhythmic agent lidocaine (Botta et al. 1988). The local anaesthetic effect of phenol is used in peeling creams too (Ruiz-Maldonado 1997). Although acute phenol exposure can lead to severe symptoms, once recovered, long-term effects are not expected to appear (Vearrier et al. 2015). Phenol peel is used today, but the protocols are strict and the applied products have lower side effect profile (Park et al. 2007). In modern medicine, phenolics are also present as technological materials or standalone active substances. The latest implemented Pharmacopoea Hungarica VIII contains monographs on phenol, cresol and resorcinol used for medicinal purposes (Pharmacopoea Hungarica VIII 2003). The Formulae Normales VII (official magistral recipe collection of Hungary) contains three types of antiseptic and antifungal topical solutions, called Solutio Castellani (boric acid, phenol, resorcinol in ethanol solution) (Formulae Normales VII 2003). The other well-known property of phenol is the local anaesthetic effect. In otolaryngology, phenol has been used to anaesthetize the tympanic membrane since 1907. This method is questionable because of possible damage to the tympanic membrane and the cochlea, as phenol exerts neurotoxic effect and a potential hazard for sensorineural hearing loss (Robertson et al. 2006). Medicine also uses phenol to treat haemorrhoids. Liquefied phenol content of ointments absorbs well and rapidly from the skin. Acting on the sensory nerves, it causes numbness and decreases itching sensation. Injection sclerotherapy for haemorrhoids involves 5% phenol dissolved in almond oil (PAO). Potassium aluminium sulphate and tannic acid (ALTA) are used as additives (Yano and Yano 2015; Acheson and Scholefield 2008). A classical usage of phenolics is to treat warts and verrucas (Dalimunthe et al. 2018). Several traditional non-official magistral medicine contained phenol, cresol, resorcine and salycilic acid, mainly dissolved in aether or collodion (aetheric solution of cellulose nitrate). In today’s medicine, these medicines are sporadic. Salicylic acid replaced phenolics. Despite the traditional usage of phenolics to treat warts, Kwok et al. excluded 80% phenol from reviewing, because according to the collected trials, the treatment showed no advantage over placebo (Kwok et al. 2012). Percutaneous neurolysis is a way to manage pain especially among cancer patients. A chemical agent (usually 3–20% phenol or absolute ethanol) is injected in the corresponding area of the sympathetic plexus or peripheral nerves. During chemical neurolysis, the chemicals cause sensory nerve damage, thus blocking the nociceptive signals (Filippiadis et al. 2009). The chemicals cause Wallerian degeneration, which is a controlled self-destruction of the axons; the axon is dying back (Coleman and Freeman 2010). Another possible mechanism of action might be the ryanodine receptor (RyR) modulation of phenolics. Ryanodine receptors have an important role in the regulation of intracellular calcium levels in the nervous system and muscle. RyR has several well-known chemical modulators. Some pharmacons act as activators (caffeine, halothane), while others exert inhibitory effect (neomycin). A well-known activator of RyR is 4-chloro-m-cresol (4-CmC) (Van Petegem 2012). Not only does this halogenated phenolic compound activates RyR but also other phenolics are able to exert partial or full activation as well. Jacobson et al. studied several structural analogues of 4-CmC concerning their ability to activate RyR1. In vitro binding and in vivo cell line analyses were used to test the analogues. They concluded that the hydroxyl group at position 1 is essential. Several other substituents were also tested. Among these substituents 3,4-dimethilphenol (3,4-xylenol) was an agonist with smaller activation potential than 4-CMC (Jacobson et al. 2006). In the aforementioned analytical studies of thermal waters, 3,4-dimethilphenol was present. There are several other types of phenolic compounds reported as phenol derivatives too (Fekete 2013; Kompanichenko et al. 2016). These compounds might have the same effect on RyR1.

## Discussion

As BTEX compounds and other alkyl benzenes are considered toxic and environmental pollutants, it should be mentioned that they are hazardous chemicals that might have a toxicological effect during balneotherapy. For this reason, several balneotoxicological studies were conducted (Gerencsér et al. 2010; Szendi et al. 2012). Medicinal waters contain these chemicals. During balneotherapy, people are exposed to them by dermal absorption and inhalation or oral exposure. As many inhalant abusers consume the same chemicals, we can hypothesise that people who underwent balneotherapy with waters containing high BTEX and alkylbenzene suffer a low-dose abuse of these chemicals. The dose is lower than in the case of solvent abuse. However, in the case of a full-day exposure, where most of the time the person submerges in medicinal water and inhales the vapours all day, these exposures are cumulating. Glutaminerg NMDA receptors can be found in the spinal cord. They are in connection with nociceptive neurotransmission. In persistent pain states, central sensitisation is important. Glutaminerg NMDA receptors are involved in this process (Das 2015). Taking into account that benzene acts on these receptors, it might be involved in chronic pain as well. Simple phenolics in medicine are used in higher concentration than that of medicinal waters. We do not know the proper pharmacokinetics. We can suppose that the absorbed simple phenolics act as local anaesthetics and thus contribute to the effect of medicinal waters in painful musculoskeletal disorders. At least one compound is present in phenolic medicinal waters that have an agonist effect on RyR1 (3,4-dimethylphenol). The ryanodine receptor calcium release channel that can be found in several organs including skeletal muscles, CNS and the heart. It is responsible for many different functions (Dulhunty et al. 2018). Excess Ca2+ release from RyR is in connection with muscle weakness in rheumatoid arthritis (Yamada et al. 2017). RyR is in connection with wound healing. RyR antagonist dantrolene positively affected wound healing in mouse wound healing models (Degovics et al. 2019). RyR modulates several biochemical pathways and is able to react to chemicals from the inflammation cascade. The precise role of RyR in inflammatory response has not been elucidated yet (Buck and Ehrlich 2005). It is notable that antagonists of the RyR are able to exert positive health effects (Hopp et al. 2015). 3,4-Dimethilphenol is an agonist of the receptor. The possible treatment of cutaneous warts by medicinal water is rooted from case reports and long-time experience. Gülec et al. investigated a small number of organ transplant patients with thermal water and heated tap water. They achieved negative results, but concluded that different types of thermal water should be tested for the same purpose (Güleç 2018).
